# IS LAPAROSCOPIC REOPERATION FEASIBLE TO TREAT EARLY COMPLICATIONS AFTER LAPAROSCOPIC COLORECTAL RESECTIONS?

**DOI:** 10.1590/0102-672020190001e1502

**Published:** 2020-07-08

**Authors:** Rodrigo Ambar PINTO, Leonardo Alfonso BUSTAMANTE-LOPEZ, Diego Fernandes Maia SOARES, Caio Sergio R. NAHAS, Carlos Frederico S. MARQUES, Ivan CECCONELLO, Sergio Carlos NAHAS

**Affiliations:** 1 Department of Gastroenterology, Hospital das Clínicas, School of Medicine, University of São Paulo, São Paulo, SP, Brazil

**Keywords:** Laparoscopy, Colorectal surgery, Postoperative complications, Laparoscopia, Cirurgia colorretal, Complicações pós-operatórias

## Abstract

***Background*:**

Recently, with the performance of minimally invasive procedures for the management of colorectal disorders, it was allowed to extend the indication of laparoscopy in handling various early and late postoperative complications.

***Aim*:**

To present the experience with laparoscopic reoperations for early complications after laparoscopic colorectal resections.

***Methods*:**

Patients undergoing laparoscopic colorectal resections with postoperative surgical complications were included and re-treated laparoscopically. Selection for laparoscopic approach were those cases with early diagnosis of complications, hemodynamic stability without significant abdominal distention and without clinical comorbidities that would preclude the procedure.

***Results*:**

In four years, nine of 290 (3.1%) patients who underwent laparoscopic colorectal resections were re-approached laparoscopically. There were five men. The mean age was 40.67 years. Diagnoses of primary disease included adenocarcinoma (n=3), familial adenomatous polyposis (n=3), ulcerative colitis (n=1), colonic inertia (n=1) and chagasic megacolon (n=1). Initial procedures included four total proctocolectomy with ileal pouch anal anastomosis; three anterior resections; one completion of total colectomy; and one right hemicolectomy. Anastomotic dehiscence was the most common complication that resulted in reoperations (n=6). There was only one case of an unfavorable outcome, with death on the 40^th^ day of the first approach, after consecutive complications. The remaining cases had favorable outcome.

***Conclusion*:**

In selected cases, laparoscopic access may be a safe and minimally invasive approach for complications of colorectal resection. However, laparoscopic reoperation must be cautiously selected, considering the type of complication, patient’s clinical condition and experience of the surgical team.

## RESUMO

***Racional*:**

A realização de procedimentos minimamente invasivos para o manejo de distúrbios colorretais, possibilitou ampliar a indicação de laparoscopia para o manuseio de diversas complicações pós-operatórias precoces e tardias.

***Objetivo*:**

Apresentar a experiência com reoperações laparoscópicas para complicações precoces após ressecções colorretais laparoscópicas.

***Métodos*:**

Foram incluídos pacientes submetidos a ressecções colorretais laparoscópicas que apresentaram complicações cirúrgicas no pós-operatório abordadas por via laparoscópica. Os pacientes selecionados foram aqueles com diagnóstico precoce de complicações, estabilidade hemodinâmica sem distensão abdominal significativa e sem comorbidades clínicas que impedissem o procedimento.

***Resultados*:**

Em quatro anos, nove de 290 (3,1%) pacientes submetidos a ressecções colorretais laparoscópicas foram reabordados pela mesma via de acesso. Havia cinco pacientes do sexo masculino e idade média foi de 40,67 anos. Os diagnósticos de doença primária incluíram adenocarcinoma (n=3), polipose adenomatosa familiar (n=3), colite ulcerativa (n=1), inércia colônica (n=1) e megacólon chagásico (n=1). Os procedimentos iniciais incluíram quatro proctocolectomias totais com anastomose íleo-anal em bolsa ileal; três ressecções anteriores; uma totalização de colectomia total; e uma hemicolectomia direita. A deiscência da anastomose foi a complicação mais comum que resultou em reoperação (n=6). Houve apenas um caso de desfecho desfavorável, com óbito no 40º dia da primeira abordagem após complicações consecutivas. Os demais casos tiveram desfecho favorável***.***

***Conclusão*:**

Em casos selecionados, o acesso laparoscópico pode representar alternativa de abordagem segura e minimamente invasiva para complicações da ressecção colorretal. No entanto, a reoperação laparoscópica deve ser cuidadosamente selecionada, considerando o tipo de complicação, a condição clínica do paciente e a experiência da equipe cirúrgica.

## INTRODUCTION

Reoperation is defined as a surgical intervention to solve complications after the initial procedure, early or delayed. Late or delayed re-intervention usually happens months or years after the initial procedure. More likely, this type of reoperation does not have the intention to repair any mistake performed before. In this case, the previous intervention might be a challenge for the performance of the reintervention, due to adhesions or other difficulties.

Early reinterventions are in the present report classified as reoperations, as they aim to repair a complication of the initial colorectal surgery. The main indications for reoperation in colorectal surgery are: anastomotic leak, intra-abdominal or pelvic abscess, bowel obstruction, ureteral and bowel injury, vessel bleeding, and others. Anastomotic leak is the most important complication due to its morbidity and mortality. The incidence of stoma formation after anastomotic leak varies from 10-100% inducing to long permanence at the intensive care unit, severe sepsis, wound infections and other abdominal wall complications^3^ ^,^ ^6^ ^,^ ^19^.

Reoperations, even after laparoscopic colorectal procedures, are traditionally approached laparotomically. Many surgeons consider peritonitis as a contra-indication to laparoscopy due to increased sepsis risk from the pneumoperitoneum, higher risk of injury of the distended bowel, better view of the abdominal structures and better irrigation through an open approach^9^ ^,^ ^16^ ^,^ ^24^. However, some other authors have shown in case series the feasibility and benefits of laparoscopic approach to early complications, showing potential lower risks of abdominal wall complications and better postoperative recovery after minimally invasive procedure^14^ ^,^ ^20^.

Potential technical advantages of laparoscopy for reoperation include the fact that the trocars can be bluntly reinserted in the abdominal cavity and the pneumoperitoneum obtained as an open manner to reduce the risk of bowel injury. The reoperation, on the other hand, need to be as early as possible, to avoid contamination and bowel distention. The late surgical indication of reoperation might preclude the approach due to gross contamination, diffuse bowel distention and adhesions. Surgeon experience in laparoscopic approach is another important factor, as an inexperienced surgeon might increase the risk of unexpected injuries that could put the patient in higher morbid condition.

Recently, the increased experience with minimally invasive colorectal procedures extended its indication to early and late complications. However, there is a lack of experience with this approach for reoperation in the literature to confirm the hypothesis that laparoscopy is better than laparotomy also for reoperation.

The aim of this study was to present the experience with laparoscopic reoperations for early complications after laparoscopic colorectal resections and compare with patients approached conventionally.

## METHOD

### Patients

After institutional review board approval (number 9076078) a retrospective evaluation of a prospectively collected database of patients undergoing colorectal laparoscopic resections was made. Patients with early postoperative complications approached laparoscopically were elected. The selection criteria for laparoscopy (lap group) included: early diagnosis, hemodynamic stability, no significant abdominal distention, no significant co-morbidities that preclude the procedure through laparoscopy. A group of patients who underwent open reoperation (open group) was used for comparison with the patients approached laparoscopically.

### Operative technique for laparoscopic reoperation

Patients were under general anesthesia, with nasogastric tube and split legs. Skin and aponeurotic stitches were removed and the first umbilical trocar is bluntly inserted under direct vision inside the abdominal cavity. Pneumoperitoneum was slowly introduced to prevent damage and the camera was inserted through the trocar for a laparoscopic diagnosis. If there was no important abdominal distension or gross contamination to deny the laparoscopic procedure, other 2-3 previous trocar incisions would be reused to insert new trocars and perform the procedure.

The procedure consisted in identify the problem by screening all small and large bowel and solve as fast as possible, followed by cavity washout with saline solution and drainage. Loop ileostomy as well as reopening the Pfannenstiel was performed only on demand. Anastomotic leaks were sutured if possible; otherwise, the leak region was well drained, and always a distal rectal saline lavage was performed under laparoscopic view to clear the anastomosis from gross contamination.

At the end of the procedure the trocars were removed under direct vision, incisions closed and the patient allocated in intensive care unit.

### Parameters analyzed

Preoperative parameters reviewed were age, gender, patient’s primary disease and type of complication for surgical indication. Operative parameters included operative time in minutes and stoma construction. Postoperative parameters were length of hospital stay in days and mortality.

### Statistical analysis

SPSS 20.0^®^ was used for data comparison. Descriptive data were expressed as mean±standard error of the mean, or as the number of patients and the percentage. Unpaired t test was applied for the analysis of ordinary quantitative data and proportional z test for the analysis of quantitative positive events. Statistical significance was indicated when the p value was less than 0.05.

## RESULTS

Nine of 290 (3.1%) patients who underwent laparoscopic colorectal resections were reproached by laparoscopy and included in the study. In the same period, 15 (5.17%) underwent an open reoperation after laparoscopic colorectal resections and were used as a control group.

The mean age for the lap group was 40.67 (±13.1) years and the proportion of male/female was comparable (five men and four women). The open group had a mean age of 63.7 (±10.65) years and the same gender proportion (eight men and seven women).

Primary disease in the lap group was colorectal adenocarcinoma (44.4%), followed by familial adenomatous polyposis (33.3%), mucous ulcerative colitis (11.1%), colonic inertia (11.1%) and Chagas’ megacolon (11.1%). The open group had 10 (66.7%) patients with adenocarcinoma, three (20%) with adenoma, one (6.7%) with Chagas’ megacolon and one (6.7%) with adenomatous polyposis. The initial procedures as well as the reason for reoperation are described in Table 1.

Of the procedures performed during lap reoperations seven used only the previous trocars without opening incision to help wash the abdominal cavity, suture dehiscence point, drainage and externalization of a stoma protection. Two reoperations required prior incision reopening to wash the abdominal cavity and exteriorization of terminal colostomy in one case, and suture of inadvertent injury in the terminal ileum in another case. Postoperative outcomes were satisfactory after reoperation in eight patients, who had an average total hospital stay of 14.6 days and 9.9 days after reoperation. One had an adverse outcome after the first laparoscopic reoperation, reoperated four times by laparotomy, and died from abdominal surgical site infection 40 days after the first procedure, which had been an anterior rectal resection for rectal carcinoma. The mean operative time for the lap group was 56.4 min (±9.15).

**TABLE 1:** Initial procedures and reasons for reoperation

Inicial procedure	n	Reoperation cause
Lapgroup (n=9)		
Total proctocolectomy + IPAA	4	2 anastomotic leaks 1 small bowel obstruction 1 omental bleeding vessel
Anterior resection	3	3 anastomoticleaks
Completionof total colectomy	1	Anastomoticleak
Right colectomy	1	Accidental ileal injury
Open group (n=15)		
Anterior resection	9	4 anastomotic leaks 3 small bowel obstructions 2 accidental small bowel injury
Right colectomy	4	2 small bowel obstructions 2 accidental small bowel injuries
Total proctocolectomy + IPAA	1	Anastomotic leak
Abdomino-perineal resection	1	Ureteral injury

Open reoperations were performed through a midline trans-umbilical incision to perform the necessary repairs, wash the abdominal cavity and have a stoma done when needed. Operative and postoperative analyses are described in Table 2.

**TABLE 2:** Operative and postoperative data

Data	Lapgroup (n=9)	Open group (n=15)	p
Operative time (min)	56.44 (±9.15)	74.8 (±16.65)	0.001*
Stoma confection	4 (44.45%)	7 (46.67%)	0.95
Overall hospital stay (days)	14.62 (±5.29)	15.18 (±7.9)	0.41
Hospital stay after reoperation (days)	9.87 (±4.97)	12 (±6.42)	0.19
Mortality	1(11.1%)	4 (26.67%)	0.03┼

*=t test; ┼=proportion z test

## DISCUSSION

Operative morbidity related to laparoscopic resection colorectal varies in the literature from 20-40%^13^ ^,^ ^15^ ^,^ ^18^ ^,^ ^22^ ^,^ ^25^. Previous studies with gastric surgery and cholecystectomies have demonstrated the benefits of the laparoscopic reapproach as well as late laparoscopic reapproach after open surgery has also proved safety and feasible in other scenarios^13^ ^,^ ^17^ ^,^ ^21^ ^,^ ^23^.

The main advantage of laparoscopic access for reoperation is the fact that, despite the existence of a complication, the patient still benefits of minimally invasive access^20^.

The present study shows that laparoscopic reoperation for early complications after laparoscopic colorectal resection is feasible and can be safe with regard to conversion, perioperative morbidity, operative time and abdominal wall preservation for the requirement of further incisions.

The increasing experience of laparoscopic surgery in urgency and emergency settings has enabled the approach of critically ill patients, even in the presence of peritonitis. Reoperation for dehiscence of colorectal anastomosis is usually approached conventionally, mainly due to intestinal distension that can limit the surgeon vision, increasing the risk of inadvertent injury. Sauerlandet al^20^ observed that trained and experienced surgeons can achieve diagnostic and therapeutic accuracy greater than 90%^18^. Compared to conventional surgery, laparoscopy has the advantage of reaching all areas of the abdomen with the scope, which sometimes, cannot be done by open surgery.

Early reoperation have the advantage of trocar introduction directly through prior unhealed trocar incisions to achieve open pneumoperitoneum, as previously shown by Wind et al and performed in our cases^10^ ^,^ ^27^. This technique minimizes the risk of intestinal lesions, even in patients with intestinal distension.

Recovery after laparoscopic colorectal surgery tends to occur earlier compared to the conventional approach^1^ ^,^ ^26^. Anastomotic leakage should be suspected in cases where the patient has clinical evidence of sepsis associated with poor oral intake. Thus, the second intervention tends to be earlier in laparoscopic surgery, also an effective diagnostic tool for the management of complications while minimizing morbidity to the patient. Early reoperation minimizes the risk of septicemia and generalized peritonitis. On the other hand, chronic peritoneal sepsis with seepage and fibrin in the peritoneal cavity may be an obstacle for laparoscopic access.

During the development of the laparoscopic approach some studies emphasized the adverse effects of pneumoperitoneum on peritoneal contamination, claiming that increased intra-abdominal pressure would result in toxemia and sepsis^8^. New studies, however, found that actually open surgery altered the immune response by modifying the levels of circulating cytokines and impairs the cellular response. Moreover, the function of macrophages is better preserved by laparoscopy. Other authors also suggest that laparoscopy may be beneficial in the management of intra-abdominal sepsis, resulting in less morbidity and postoperative sepsis^9^ ^,^ ^14^. Another advantage of the laparoscopic approach, not only for reintervention, is less abdominal wall complications such as evisceration, wound infection and incisional hernias.

Two previous studies have compared laparoscopic and open reintervention. Wind et al.^27^ comparing 10 patients reoperated through laparoscopy with 15 approached conventionally, found benefits favoring laparoscopy such as shorter hospital stay and less need for intensive care unit postoperatively. Rotholtz et al.^18^ who compared 17 patients reoperated laparoscopically and 10 by conventional approach, found no such advantages justifying the similar findings due to the small sample, despite a tendency to reduction of hospital stay in the laparoscopic patients. It is noteworthy that both series were retrospective and that the cases were previously selected for each kind of approach. In this study, there was a selection of patients with early diagnosis, hemodynamic stability, no significant abdominal distention, no significant co-morbidities for laparoscopic reoperation, which could be a selection bias for the better results obtained in this group of patients.

One of the most frequent complications of colorectal surgery is anastomotic dehiscence, with an incidence ranging from 1-30%, with ideal values between 2-5%^2^ ^,^ ^6^ ^,^ ^11^ ^,^ ^15^ ^,^ ^19^. Mortality associated with this complication ranges from 25% to 50%. In the present study, as in similar studies previously published, the anastomotic dehiscence was the most common reapproach indication (66.7%). In cases of viable anastomosed colon and a small point of dehiscence, comprising 25% or less of the anastomosis, was preferred to wash the abdominal cavity associated with the local repair stitches, drainage and protective ileostomy. If the dehiscence had higher proportions or there was doubt in the anastomotic vitality, the option was to undo the anastomosis and exteriorize a terminal stoma, in addition to the procedures previously described. Recently, Kwaket al^12^ studied the reapprochement to the anastomotic dehiscence for colorectal cancer in 72 patients, and in 26 of them opted for laparoscopy. The only statistically significant data supporting the laparoscopic approach was the lowest index of wound infections (3.8% vs. 25.8%, p=0.0031). Other data, such as hospital stay and general complications were similar between groups. There was a tendency to earlier oral intake in patients re-operated by laparoscopy (5 vs. 6 days, p=0.057). Similarly, the present study had comparable hospital stay and postoperative morbidity between the two groups, although the mortality in the laparoscopic group achieved statistic significance due to the number of patients. In addition, the operative time was significantly shorter in the lap group, probably due to the faster way to get to the problem and the longer time to close the abdominal wall in the open group.

This study presents several limitations. It is a case series of early complications of laparoscopic colorectal resections reoperated through the same approach in a single center retrospectively analyzed. The absence of an adequate control group for comparison does not permit consistent conclusions. On the other hand, the literature around this specific topic is still very inconsistent, including retrospective case series and limited number of patients. New prospective comparative studies between open and laparoscopic reapprochement, preferably multicentric and randomized, would be necessary to confirm the favorable outcomes found to date.

## CONCLUSION

This preliminary study suggests that in selected cases, laparoscopic access may be a safe and minimally invasive approach for complications of colorectal resection. However, laparoscopic reoperation must be cautiously selected, considering the type of complication, patient’s clinical condition and experience of the surgical team.
